# Assessment of pre-, peri-, and post-surgical practices for elective colorectal patients in a model 4 hospital in Ireland

**DOI:** 10.1007/s11845-024-03731-4

**Published:** 2024-06-08

**Authors:** Gavin David O’Connor, Róisín Taplin, Clodagh Murphy

**Affiliations:** 1 University Hospital Limerick, Limerick, Ireland; 2 University College Cork, Cork, Ireland

**Keywords:** Anaesthesia, colorectal surgery, enhanced recovery after surgery, ERAS

## Abstract

**Introduction:**

The ERAS protocol is a set of international guidelines established to expedite patients’ discharge after colorectal surgery. It does this by aiming to prevent postoperative complications early, and return the patient to normal function allowing earlier discharge. Complications such as PONV, DVT, ileus and pain are common after surgery to name a few, and delay discharge. Early treatment and prevention of these complications however is suggested to aid a patients’ return to home at earlier rates than traditional practice.

**Methods:**

A prospective chart review and questionnaire was performed on patients undergoing colorectal surgery in UHL in a 6-month period from February to September 2023. Patients were approached on the 3rd day postoperatively and informed about the project. Exclusion criteria included patients who went to HDU or ICU postoperatively.

**Results:**

In total, 33 patients were recruited. A target of greater than 70% compliance was reached for a variety of the elements of the ERAS protocol such as laparoscopic surgery, preoperative assessments, nutritional drinks, LMWH, oral intake within 24 h of surgery, and intraoperative antiemetics. Unsatisfactory compliance was found with documentation of postoperative antibiotics use of preoperative gabapentin.

**Conclusion:**

UHL has a satisfactory compliance of over 70% with a large variety of elements of the ERAS protocol. Areas of improvement required include postoperative antibiotic and preoperative gabapentin usage. With the collective effort of the multidisciplinary team, along with education, the ERAS protocol can successfully be applied and implemented in a model 4 hospital in Ireland.

## Introduction

### Enhanced Recovery After Surgery Protocol

Enhanced recovery after surgery (ERAS) is a protocol developed in 1997 with the aim of expediting discharges in patients undergoing colorectal surgery [1]. The ERAS protocol outlines a variety of measures that should be taken throughout a patients’ surgical stay, including at the first meeting in the preoperative phase [2]. The protocol includes preoperative, perioperative and postoperative measures to be followed, with the primary objective of getting patients fit for discharge, without compromising patient care. The ERAS protocol aims to promptly treat, and prevent, common postoperative complications that increase morbidity, mortality, or length of stay in patients. Such complications include, but are not limited to postoperative nausea and vomiting (PONV), deep vein thrombosis (DVT), pulmonary embolism (PE), ileus, fluid overload and pain [2]. The ERAS protocol is often viewed as the gold standard in terms of patient care for those undergoing elective colorectal surgery.

The ERAS protocol is a large set of guidelines, and requires the collaboration of a multidisciplinary team. Preoperative measures include adequate preoperative assessment, patient education, nutritional assessment, and preoperative carbohydrate drinks. Perioperative factors include preoperative antibiotics, PONV prophylaxis, nerve blocks, and adequate fluid replacement. Postoperative factors include early mobilisation, early return to eating and drinking, removal of catheters and the use of prophylactic anticoagulants [2]. These are a wide set of recommendations to be incorporated that require the collaboration of surgeons, anaesthesiologists, nursing staff, physiotherapists and dieticians, among others, to perform.

### Inpatient stay after colorectal surgery

Colorectal surgery is common, with over 600,000 operations occurring in the United States per year [3]. It can be performed for a variety of aetiology, including colorectal cancer resection. The patient demographic undergoing colorectal surgery are often over 65 years of age with multiple comorbidities [4]. Median length of stay (LOS) of patients undergoing elective colorectal surgery has been reported in some studies to be 14 days [5]. With such a large number of patients undergoing colorectal surgery each year, and a significant inpatient stay, factors which can expedite a patients’ return to function and discharge are very valuable both in terms of their experience, and financial savings to the patient and hospital.

The ERAS protocol has been shown through a variety of research to enhance early discharge, whilst not increasing 30-day readmission rates [6–11]. It has been suggested that the 30-day readmission rates can decrease from 19% pre-ERAS to 12% after ERAS [9]. Strong data has been published that greater compliance to the ERAS protocol decreases a patients’ LOS [7–9, 12], and reduces postoperative complications and readmissions from 59.2% pre-ERAS to 34.1% post-ERAS [12]. Compliance can improve greatly over time as institutions continue to implement ERAS into their practice [7]. With all of these benefits in mind, we wanted to assess to current compliance of the ERAS protocol in UHL in patients undergoing elective colorectal surgery in 2023.

### Study aims

To assess the pre-, peri-, and post-surgical practices for patients undergoing elective colorectal surgery in a mode 4 hospital in Ireland in 2023.

### Study objectives

Primary objective: Comparing the current pre-, peri-, and post-surgical practices for patients undergoing colorectal surgery in University Hospital Limerick with the enhanced recovery after surgery protocol.

## Methods

### Study design and participants

This study is a prospective cohort study of all patients in University Hospital Limerick, Ireland who underwent elective colorectal surgery from February to September in 2023. The theatre lists of the colorectal surgeons was accessed during the study period to identify patients who were having elective colorectal surgery for inclusion in our study. Inclusion criteria included patients who were over the age of 18, undergoing elective colorectal surgery, who were not admitted to a critical care bed (ICU or HDU) postoperatively, who were an inpatient day 4 postoperatively, had capacity to consent to be in a study, and consented to participate in the study. Exclusion criteria included patients under the age of 18, not undergoing elective colorectal surgery, admitted to a critical care bed postoperatively, no longer an inpatient, lacking capacity, or declined to participate in the study. In total, 33 patients agreed to participate in the study, who granted the investigators access to their medical charts, and filled out a short questionnaire.

### Study measures

Variables to be collected were taken from the ERAS guideline to assess the care provided to patients in UHL in comparison to the standards set out in the ERAS guideline. Variables collected included age, sex, name of procedure, anti-emetics used, MUST score, preoperative weight, preoperative nutritional drink provided, date of surgery, postoperative weight, weight change, PONV, date of discharge, length of stay, postoperative complications, preoperative antibiotics, prophylactic antibiotics, intraoperative fluid type, pre-operative gabapentin, time of first oral intake, diet day 0 to day 3, day of first bowel motion, DVT prophylaxis, physiotherapy interaction, elective nasogastric tube use, attendance at preoperative assessment clinic and fasting status prior to the operation. Satisfactory adherence to the ERAS protocol was defined as compliance of 70% or greater in accordance with published literature.

### Ethical approval

Ethical approval to conduct this study was sought from the Ethics Committee in University Hospital Limerick on 23rd of September, 2022. Ethical approval was granted on 29th of November 2022.

### Data analysis

All of the data collected was entered into IBM SPSS version 22.0 for Windows (SPSS, Chicago, Illinois, USA) for analysis. Continuous variables were analysed using descriptive statistics to calculate the mean, median, range and 95% confidence intervals. Categorical variables were analysed using frequency tables.

## Results

### Study cohort

The total sample size was 33 patients with a male predominance (Table 1). The median age for patients included in the study was 70 years, mean age was 66.03 (standard deviation, 11.92, range 35 to 84): 65.2 for men and 67.4 for women. Length of stay ranged from 6 to 22 days with a mean of 11 (SD 3.886, median 10).

**Table 1:** Patient Demographic

Cohort	Result
Age (years)	66 ± 11.92
Female, n (%)	13 (39.4)
Length of stay	10.97 ± 3.886

Data shown as % of all patients or mean ± standard deviation

### Compliance preoperatively

In this study, 100% of patients attended the preoperative assessment clinic (POAC). During this clinic, 100% of patients were supplied with fasting guidelines for food and water to follow before their surgery, and had their starting weight documented. The Malnutrition Universal Screening Tool (MUST) was used on 72.7% of patients. Only 24.2% of patients drank any water on the morning of surgery, despite the guidelines saying that they must only fast for water 2 h prior to surgery. Preoperative gabapentin was used in 48.4% of patients (Table 2).

**Table 2:** Preoperative criteria of ERAS protocol

Preoperative criteria	Total, n (%)
Attended POAC	33 (100)
MUST score	24 (72.7)
Water morning of surgery	8 (24.2)
Preoperative gabapentin	16 (48.4)
Preoperative nutrition drink usage	32 (97)
Fasting guidelines supplied	33 (100)
Preoperative weight documented	33 (100)

Data shown as % of all patients; POAC, Preoperative assessment clinic;MUST, Malnutrition Universal Screening Tool

### Compliance perioperatively

Laparoscopic surgery techniques were used on 97% of patients who underwent elective colorectal surgery in UHL. No patient had a nasogastric tube leaving the operating theatre. Single anti-emetics were used in 78.8% of patients, with 21.2% of patients receiving dual anti-emetics. Granisetron was used in 69.7% of patients. CSL was the IV fluid used in 75.8% of cases (Table 3).

**Table 3:** Perioperative criteria of the ERAS protocol

Perioperative criteria	Total, n (%)
Laparascopic surgery	32 (97)
Nasogastric Tube leaving theatre	0 (0)
Granisetron	23 (69.7)
Single anti-emetic	26 (78.8)
Dual anti-emetic	7 (21.2)
CSL	25 (75.8)

Data shown as % of all patients;CSL, Compund Sodium Lactate

### Postoperative compliance

Patients were mobilising with a physiotherapist within 24 h after surgery in 100% of cases in this study. Low molecular weight heparin (LMWH) was initiated in 51.5% of patients on day 0 and 100% of cases on day 1 postoperative for DVT prophylaxis. Antibiotic prophylaxis was continued for 24-h in 90.1% of patients. Postoperative complications were recorded in 57.6% of patients, including 30.3% of patients experiencing an ileus, 12.1% of patients being treated for a lower respiratory tract infection (LRTI). Surgical site infections were recorded in 2 patients, with 1 patient experiencing notable pain, 1 patient requiring a postoperative blood transfusion, and 1 patient experiencing a surgical complication. In our study, mean LOS for patients with an ileus was 13.13 days (range 9 to 22 days, SD 4.155) compared to 10.22 in those without an ileus (range 6 to 18 days, SD 3.58). No patients experienced a DVT or PE. In this study, 33.3% of patients had oral intake on the same day after their surgery, whilst 93.9% had oral intake day 1 postoperatively, and 100% having oral intake day 2 postoperatively. Postoperative weights were only recorded in 48.5% of patients. First bowel motion had a mean of 2.76 days (SD 1.953) (Table 4).

**Table 4:** Postoperative criteria of the ERAS protocol

Postoperative criteria	Total, n (%)
Physiotherapy within 24 h	33 (100)
LMWH day 0	17 (51.5)
LMWH day 1	33 (100)
24-h prophylactic antibiotics	30 (90.1)
Complications	19 (57.6)
Ileus	10 (30.3)
LRTI	4 (12.1)
DVT/PE	0 (0)
Post-operative weight	16 (48.5)
Eating day 0	11 (33.3)
Eating day 1	31 (93.9)
Eating day 2	33 (100)

Data shown as % of all patients; LMWH, Low Molecular Weight Heparin; LRTI, Lower Respiratory Tract Infection; DVT, Deep Vein Thrombosis; PE, Pulmonary Embolism

## Discussion

In terms of preoperative aspects of the ERAS protocol, our adherence in the hospital was satisfactory at over 70% for almost all aspects. All patients had attended the preoperative assessment clinic, where they were reviewed by the surgical team and preoperative assessment nurses. This is an important clinic to get patients optimised for surgery [13]. Potential issues in relation to the surgery and anaesthetic are highlighted at this clinic. Decisions are made if patients require extra preoperative tests, such as chest x-rays or echocardiograms. Some patients require the review of specialists prior to surgery, should they have heart, lung, kidney or endocrinological co-morbidities. Potentially complicated patients are highlighted to the anaesthetic team at this clinic, who may guide and request specific investigations so that a safe anaesthetic can be performed [14].

At this clinic, many tasks were carried out. Practical tasks such a routine preoperative bloods and ECGs were performed, weights were measured and documented, guidelines were given with regard to which medications to withhold the day of surgery and the days before, along with fasting guidelines for food and water preoperatively. A nutritional drink was also supplied, which is a carbohydrate rich drink shown to improve blood sugar control postoperatively, and may also reduce PONV [15]. The MUST score was also measured and documented to highlight patients who are malnourished and may benefit from the review of a dietitian. Malnourishment has been associated with decreased healing potential and increased postoperative complications and LOS [13]. All of these factors had a satisfactory compliance of over 70%. Whilst patients had been given fasting guidelines not to drink water 2-h before their operation, over 75% of patients did not drink any water on the morning of surgery. This is likely out of fear of cancellation or delaying their surgery, despite being permitted in the guidelines. Preoperative gabapentin loading is used as an adjunct to control postoperative pain [16]. It was used in only 48% of patients in our study.

With respect to intraoperative factors investigated, adherence to the ERAS protocol was satisfactory for numerous aspects. Laparoscopic surgery was used in 97% of cases. This has been shown repeatedly in studies to aid recovery after colorectal surgery, and reduce the LOS [9, 10]. The benefits of laparoscopic surgery versus open surgery are numerous when performed by a skilled and competent operator and include reduced pain, earlier mobilisation, reduced complications and reduced LOS [11, 17]. Open surgery and stoma formation however have been associated with increased LOS after elective colorectal surgery [6]. Other features of the ERAS protocol associated with decreased LOS included intraoperative warming, early cessation of IVF and early removal of catheters and NG tubes [18], although conflicting research has been published with respect to laparoscopic surgery and fluid restrictive strategies [11].

Intraoperative NG placement is common during colorectal surgery for a number of reasons. Nasogastric tubes can be placed to drain stomach contents reducing the risk of aspiration, which can be particularly useful when laparoscopic techniques are being utilised [19]. NGs have also been used to prevent a build-up of pressure in the bowel causing distension which can lead to wound dehiscence and anastomotic leakage [20, 21]. The ERAS protocol encourages the removal of these NGs in theatre, using them for the minimal amount of time clinically indicated. In this study, no patient left theatre with a nasogastric tube. This has important benefits in terms of enhancing patient comfort, whilst also encouraging oral intake [21].

Compound sodium lactate (CSL), also known as Hartmann’s solution, is the preferred crystalloid of choice for maintenance fluids and resuscitation fluids intraoperatively in the majority of patients, as described by the ERAS protocol. CSL is a more physiologic fluid with respect to its electrolyte content, and reduces the risk of hypernatraemia and hyperchloraemic metabolic acidosis which can be seen with 0.9% normal saline [22]. Normal saline may be preferred in specific circumstances, such as patients who are hyponatraemic or hypochloraemic [23, 24]. Adherence to this policy was over 75% in this study, highlighting how CSL has become the fluid of choice intraoperatively.

Anti-emetics are important intraoperatively to help prevent PONV. PONV is a debilitating condition associated with anaesthetic gases and drugs, and can lead to an increased inpatient stay due to reducing PO intake and leading to complications such as AKI (acute kidney injury) and surgical dehiscence [25]. Anti-emetics were given intraoperatively to all patients in this study. Single agent anti-emetics were used in 79% of patients, with 21% of patients receiving dual anti-emetics. Granisetron is the preferred anti-emetic of choice as it is advantageous over ondansetron due to its longer duration of action requiring it to be dosed only once a day versus three times a day in the case of ondansetron [26]. Granisetron was used in 69.7% of patients which was just below the satisfactory level. Dual agent anti-emetics included dexamethasone. Dexamethasone has side-effects such as increasing postoperative blood sugars, peptic ulcers, impaired wound healing, infection and surgical dehiscence [27]. Reasons that anaesthetists may have opted to omit dexamethasone are numerous and include male patients who have reduced incidences of PONV according to the Apfel score, and therefore would not require dual anti-emetics [28], elderly patients who may be more sensitive to the side-effects of dexamethasone, and patients with type 2 diabetes in whom elevated glucose levels postoperatively would be undesirable [27].

Prophylactic antibiotics are an important element of the ERAS protocol, and are also included in the WHO safe surgery checklist [29]. They should be administered within 60 min before skin incision to ensure they reach a desirable plasma concentration in the tissue before contamination with bacteria occurs as the skin is pierced [29]. Co-amoxiclav is the antibiotic of choice in our hospital in patients who are not penicillin allergic undergoing elective colorectal surgery.

Postoperative elements of the ERAS protocol were investigated extensively in this study. The discharge criteria for patients after elective colorectal surgery usually involve ensuring pain is adequately controlled, patients can mobilise independently or are back at baseline, patients can tolerate adequate oral intake, and normal bowel and bladder function have returned [30]. All of this should be accomplished without any complications before discharge.

In our hospital, patients are seen within 24-h of surgery by physiotherapists in 100% of cases. Physiotherapists are essential members of the multidisciplinary team. They benefit the care of patients by encouraging mobilisation in a safe manner after surgery which can decrease the risk of DVT and PE, whilst also providing breathing exercises to prevent complications such as atelectasis [31].

LMWH is used routinely in hospital to help prevent DVTs and PEs, which are known postoperative complications, usually associated with decreased mobility among other risk factors [32]. LMWH was administered on the same day as the surgery (day 0) in 52% of patients, with 100% of patients receiving LMWH by day 1 postoperatively. No patient had a DVT or PE in this study.

Antibiotic prophylaxis is an important element to prevent postoperative complications such as surgical site infections [33]. To prevent these complications, antibiotics should be administered for 24-h postoperatively, before being discontinued, unless there is a clinical reason to continue antibiotics for longer. Antibiotic prophylaxis in accordance with this policy was administered in 90% of patients.

Increased compliance with the ERAS protocol has been correlated with a decrease in complications postoperatively, and a reduced LOS [12]. Increased compliance has been associated with a decreased LOS [7–9], reduced postoperative complications and readmissions [12]. Enhanced ERAS compliance has also been found to reduce perioperative complications from 56% before the implementation of an ERAS protocol, to 9.4% [8]. With respect to postoperative complications in our institution, these were documented to have occurred in 58% of patients. Complications included ileus in 30% of patients, with 6% of patients having a surgical site infection, 12% having a LRTI, 3% requiring postoperative blood transfusions, 3% having significant pain, and 3% suffering from a intraoperative complication. Prolonged postoperative ileus has been reported to occur in 16.6% of patients, but has been reduced in patients who had early feeding, mechanical bowel preparation, early mobilisation, and who had laparoscopic surgery [34]. LOS in patients who had an ileus was 13 days compared to 9.5 days in those without [34]. In our study, mean LOS for patients with an ileus was 13.13 days compared to 10.22 in those without an ileus. ERAS has been shown to reduce the time to bowel function to 2.5 days from 4.1 days [35]. Our study had similar results with a mean time to bowel opening of 2.76 days.

Early commencement of oral intake is a key step in the ERAS protocol [36]. Previously, patients were routinely fasted after surgery, in an attempt to prevent complications such as postoperative ileus or dehiscence. Newer studies have disputed this practice however, and early exposure to oral intake postoperatively is associated with faster return to bowel function and regular eating habits, whilst reducing the risk of postoperative ileus among other complications [34]. In our study, 33% of patients were allowed oral intake on the same day of surgery, with 94% of patients allowed to eat day 1 postoperatively, and 100% allowed to eat day 2 postoperatively. Local policy in the hospital encourages the stepwise progression of oral intake after colorectal surgery. Sips of water are first to be introduced, followed by jelly and a high protein, fortified ice-cream to aid patients who are malnourished, whilst also providing key ingredients that aid tissue repair and recovery postoperatively [37]. Coffee is another element of the ERAS protocol that has been found to be beneficial in patients undergoing colorectal surgery, to aid the return to normal bowel function [37]. Jelly sweets and chewing gum are also encouraged to aid the return to normal bowel function [38, 39]. Following from this, tea and toast are introduced, followed by soft food such as yoghurt, eggs and potato, before commencing to a normal diet.

Whilst preoperative weight was measured in 100% of patients, postoperative weights were only measured in 49%. Postoperative weights are recommended to be taken to assess for fluid balance, with an excessive positive balance being associated with poorer outcomes such as postoperative ileus rates, and prolonged length of hospital stay [40].

The ERAS protocol has been associated significantly with existing research to decrease the LOS after elective colorectal surgery [6–9]. It has been shown that the use of the ERAS protocol does not increase 30-day readmission rates [6–8, 10, 11], whilst re-admission rates have been suggested to fall from 19% pre-ERAS to 12% post-ERAS [9]. Strong data has been published to suggests that greater compliance to the ERAS protocol can decrease a patients’ LOS [7–9, 12], and reduces postoperative complications and readmissions from 59.2% pre-ERAS to 34.1% post-ERAS [12]. Compliance can improve greatly over time as institutions continue to implement ERAS into their practice [7]. Mean LOS in patients undergoing elective colorectal surgery under the ERAS protocol has been suggested to be 4 days [11]. Our study had a much higher mean LOS of 11 with a median of 10. This was possibly due to the varying local protocol with regard to discharge criteria, along with the data collection methods which involved consenting patients who were inpatients on day 4 postoperatively. This method may have missed patients who were discharged prior to day 4, whilst also selecting patients who had suffered a postoperative complication at a rate that was potentially disproportional to the true incidence rate.

## Limitations

Ethical approval in this study only allowed patients to be approached to be informed of the study on day 3 before signing a consent form on day 4. This may have introduced bias into our study by having healthy, uncomplicated patients discharged before day 4 of their hospital stay. This may also have introduced bias as patients who underwent more extensive surgery, which naturally has a greater risk of complications, may be over represented in our study cohort, along with any patient who underwent minor surgery but suffered a postoperative complication. This may explain why our complication rate is at 58% after the implementation of the ERAS protocol, and our prolonged LOS. Patients filled out a questionnaire on day 4 of their inpatient stay. Some patients may have had difficulty remembering their oral diets and bowel movements in the days prior to this questionnaire, which may introduce recall bias. No author approached any patient that they were directly involved in the care of during this project.

## Future research

Future research based on this project should look at the various elements of the ERAS protocol that were not investigated in this project. Such elements may include the use of intraoperative blocks, such epidural catheters or transversus abdominis plane (TAP) blocks, the use of opiates and other analgesics postoperatively, and the impact of limiting opiate exposure whilst delivering analgesia on patient outcome postoperatively. Preoperative bowel prep is a topic that is suggested by the ERAS protocol to be omitted, but new research may suggest its use in clinical practice. More research on preoperative bowel prep may be required to address this topic and its possible incorporation into the ERAS protocol. Further research should investigate the efficacy of the ERAS protocol on a patient cohort who are not undergoing elective colorectal surgery, such as elective breast, urology, orthopaedic, or emergency surgery, to see if the same principles and benefits are transferrable.

## Conclusion

UHL has a satisfactory compliance of over 70% with a large variety of elements of the ERAS protocol. UHL has a successful POAC for patients undergoing elective colorectal surgery, with 100% of patients attending this clinic. Areas of improvement in UHL with regard to the ERAS protocol include preoperative gabapentin usage and postoperative antibiotic prophylaxis. No patient left theatre with an NG tube in-situ. Over 94% of patients were receiving oral intake by day 1 postoperatively, with 100% of patients mobilising with a physiotherapist day 1 postoperatively, and receiving LMWH prophylaxis. With the collective effort of the multidisciplinary team, along with education, the ERAS protocol can successfully be applied and implemented in a model 4 hospital in Ireland.

## Data Availability

Raw data if desired can be requested by contacting the lead author directly by email.
